# Love in the Time of Corona: Predicting Willingness to Engage in Sexting During the First COVID-19-Related Lockdown

**DOI:** 10.1007/s10508-022-02292-w

**Published:** 2022-02-07

**Authors:** Marina F. Thomas, Alice Binder, Jörg Matthes

**Affiliations:** 1 Department of Communication, University of Vienna, Waehringer Straße 29, 1090 Vienna, Austria; 2 Department of Media and Communications, University of Klagenfurt, Klagenfurt am Wörthersee, Austria

**Keywords:** Sexting, COVID-19, Social isolation, Privacy concerns, Loneliness, Terror management

## Abstract

When the COVID-19 pandemic began, in early 2020, lockdowns limited the options for physical intimacy and many resorted to technology-mediated forms of intimacy such as sexting. However, it is unclear what predicted willingness to engage in sexting during the lockdown. The present study filled this gap by investigating COVID-19-related social isolation, privacy concerns, age, and gender as predictors of willingness to engage in sexting. We further examined an interaction of COVID-19-related social isolation and privacy concerns on willingness to engage in sexting. We conducted online surveys with 494 young adults (Study 1) and with a quota-based sample of 437 adults (Study 2) in Austria. In both studies, negative binomial regressions revealed a positive effect of COVID-19-related social isolation on willingness to engage in sexting. Privacy concerns hindered young adults in Study 1 from engaging in sexting but not relatively older adults in Study 2. However, in neither study did privacy concerns moderate the effect of COVID-19-related social isolation on willingness to engage in sexting: Even individuals with high privacy concerns were more willing to sext under conditions of social isolation, suggesting that the need for intimacy outweighed the need for privacy protection. Gender had no effect in either study, indicating that men and women used sexting to cope with the unprecedented COVID-19-related situation.

## Introduction

In spring 2020, well over 100 countries and billions of people were in lockdown to prevent the spread of the novel coronavirus disease (COVID-19). As fear of contagion and pandemic mitigation measures forced individuals to minimize physical contact (Dunford, 2020), technology-mediated communication gained importance in satisfying the need for intimacy (Ballester-Arnal et al., 2020). One way to be intimate without physical contact is sexting, i.e., the exchange of sexually suggestive or explicit texts and (semi-)naked photographs via mobile phones and social media (Albury et al., 2013; Amundsen, 2022). Research has shown that during the first COVID-19-related lockdown, some people were willing to add sexting to their sexual repertoire (Lehmiller et al., 2021). However, we lack research on the predictors of sending sexts during the lockdown.

Due to the specific situation during lockdowns, people might experience a unique feeling of pandemic-related social isolation (Killgore et al., 2020). Sending sexts is a form of online self-disclosure, which can be a coping mechanism, according to terror management and findings on online self-disclosure (Derlega & Berg, 2013; Luo & Hancock, 2020; Mikulincer & Florian, 2000). Therefore, we expected a higher willingness to engage in sexting in individuals with increased COVID-19-related social isolation. Furthermore, since privacy concerns have been associated with decreased information sharing on social media messengers (Jozani et al., 2020), we also investigated the effects of privacy concerns and how privacy concerns moderated an effect of COVID-19-related social isolation, that is, if social isolation drove willingness to sext even in individuals with high privacy concerns.

Lastly, we tested the effects of gender (Ringrose et al., 2013) and age (Klettke et al., 2014) on willingness to engage in sexting. We tested these effects in a sample of young adults (Study 1) and a sample based on quotas regarding gender, age, and education in Austria (Study 2). Thereby, we tackled the lack of findings with regard to sexting in older adults (McDaniel & Drouin, 2015; Wiederhold, 2011).

### COVID-19-Related Social Isolation

With its growing death toll, constant media coverage, global stock market crashes, and the imminent apprehension of a collapsing welfare system, the COVID-19 pandemic spread anxiety and insecurity in early 2020 (Brooks et al., 2020; Salari et al., 2020). Social distancing measures were very strict in Austria, where there was a federal lockdown from March 16 to mid-May. Except for supermarkets and pharmacies, all restaurants, shops, and hotels were closed. Citizens were banned from public spaces and only allowed to take walks with people from the same household (up to a maximum number of five). Fines exceeded 3000 Euros. The borders to neighboring countries were shut. This situation was entirely unprecedented for Austria and it was unpredictable how long the lockdown would last (Deutsche Welle, 2021; Kleine Zeitung, 2021).

Pandemics can activate thoughts of death and thereby increase the salience of our mortality (Sole, 2007). To cope with the terror of (pandemic-based) mortality threats, humans employ different strategies. According to terror management theory, humans developed religion and other ideologies and symbolic groups so that they can be part of something enduring that outlasts their evanescent bodies (Smith & Massey, 2013). Thus, terror management theory (Greenberg et al., 1986) posits that not only directly related (“proximal”) health protection behaviors (i.e., hand washing) serve as a fear relief but also indirect (“distal”) psychological strategies such as identification with a symbolic group, cultural values (Agroskin & Jonas, 2013), or close relationships (Mikulincer & Florian, 2000).

In this regard, a common terror management and general coping strategy is intimacy (Mikulincer & Florian, 2000; Plusnin et al., 2018; Wisman & Koole, 2003). Close relationships alleviate existential threats because they validate shared worldviews, provide security, and self-esteem (Plusnin et al., 2020). Terror management theory (Greenberg et al., 1986) predicts that in contrast to animals, humans can be aware of their own transitoriness (mortality salience). When reminded of it, as a relatively distal threat, individuals may perceive an abstract existential anxiety. This worry or ontological insecurity can increase the need to belong (i.e., a need for intimacy or to cling to and defend an in-group’s worldview) as symbolic coping mechanisms. Such symbolic coping can help against distal symbolic threats but not so much against imminent fear of death or infection. In other words, sexual intimacy may not be an antidote to the concrete fear of infection but to abstract mortality salience.^1^

Feelings of intimacy can be generated through sexual contact. Yet, at the same time, physical contacts needed to be avoided to prevent COVID-19 infections. Research showed that during the lockdown, there was a sharp decrease in partnered sexual activity (Li et., 2020; Sanchez et al., 2020). In particular, those who did not live with a partner reported a decline in their sexual lives during the lockdown (Ballester-Arnal et al., 2020) Even within couples (who lived together), fear of contagion and limited opportunities have reduced physical contact (Ibarra et al., 2020; Schiavi et al., 2020) as an immediate form of stress and fear relief.

An alternative possibility to generate feelings of intimacy without physical contact are technology-mediated sexual activities. Early empirical findings confirm that during the first pandemic-related lockdown, people resorted to online sexual activities (Ballester-Arnal et al., 2020) such as pornography (Mestre-Bach et al., 2020), and dating app use (Sanchez et al., 2020). Apart from consuming sexual content of others, people might also have the desire to self-disclose and actively send intimate content to others as we theorize that the coping effect of sexting stems primarily from the self-disclosure (Luo & Hancock, 2020). Therefore, we focused on the active sending of own sexts and deemed receiving sexts from a partner or forwarding unrelated third party’s sexts irrelevant to our purposes. Moreover, we inquired future willingness and one cannot foresee how likely it is to receive sexts in the future, and reception may be unforeseen and even non-consensually (Ringrose et al., 2021).

Before the pandemic, sending sexts was prevalent in 40–50.0% of young adults (Drouin et al., 2013; Klettke et al., 2014; Mori et al., 2020) while only 13–14.8% of adolescents had sent sexts (Madigan et al., 2018; Patchin & Hinduja, 2019) and sexting is understudied in older adults (McDaniel & Drouin, 2015; Wiederhold, 2011). Research has hitherto heavily problematized sexting, even in consenting adults, and focused on negative correlates (e.g., Benotsch et al., 2013) while neglecting its psychological function: Exchanging intimate information is a vehicle for developing close relationships (Derlega & Berg, 2013) and (sexual) online self-disclosure can increases well-being (Burkett, 2015; Luo & Hancock, 2020; Parker et al., 2013). During the lockdown, health authorities even recommended sexting as a form of safer sex (International Society for the Study of Women’s Sexual Health, 2020). Research already showed that during confinement, sexting was indeed a common addition to people’s sexual repertoire, especially if they lived without a partner (Lehmiller et al., 2021).

We know from previous studies that before the pandemic, sexting was especially prevalent in physically separated couples (Le, 2016), or individuals who do not yet have an offline romantic relationship (Ševčíková et al., 2018). Generally, people report to engage in sexting to satisfy the need for closeness (Albury et al., 2013). Indeed, research showed that people generally felt socially isolated during the lockdown (Killgore et al., 2020) and that loneliness predicted exploration of novel sexual behaviors during that period (Lehmiller et al., 2021). Yet, we lack research clarifying how the specific COVID-19-related social isolation related to people’s willingness to engage in sexting.

Following the prototype willingness model of sexual risk behaviors (Gerrard et al., 2005; Gibbons & Gerrard, 1995), many studies on sexting (e.g., Schreurs et al., 2020; van Oosten & Vandenbosch, 2017, 2020; Walrave et al., 2015) or other sexual behaviors (Boot et al., 2016) focus on behavioral willingness instead of actual sexual behavior. One reason is that only three in seven individuals engage in sexting (Mori et al., 2020) but more people may be willing to if the situation presents itself. Behavioral willingness reflects the general “openness to opportunity, i.e., a willingness to engage in risky behavior in circumstances that are conducive to that behavior” (Gerrard et al., 2005, p. 306). This includes not only the volitional intention out of a dispositional motivation but also the perceived likelihood of spontaneously engaging in the behavior due to social factors. As such, willingness is a major predictor of future behavior (Gerrard et al., 2005). On the basis of the aforementioned findings (Albury, 2013; Lehmiller, 2021) and terror management theory (Greenberg et al., 1986), we hypothesized that higher COVID-19-related social isolation predicts a higher willingness to engage in sexting (*H1*).

Importantly, we expect an effect of situational COVID-19-related social isolation over and above an effect of trait loneliness. While social isolation means the absence of a social network, emotional loneliness describes the subjective unpleasant and distressing experience of lacking attachment figures (Weiss, 1973; West et al., 1986). Thus, loneliness is a rather stable personality trait (Mund et al., 2020). Research has correlated sexting with loneliness (Drouin & Tobin, 2014). However, it is important to distinguish the effects of the situational factor COVID-19-related social isolation from the effect of the personality trait loneliness. Therefore, we included trait loneliness as a control variable.

### Privacy Concerns

Exchanging intimate information generates trust and is a vehicle for developing close relationships (Amundsen, 2022; Derlega & Berg, 2013). It is the implicit norm that confidentiality is expected in a sexting context and disseminating intimate pictures without the sender’s consent is not acceptable (Hasinoff & Shepherd, 2014). However, there are cases of abuse in which untrustworthy interaction partners disseminate the highly sensitive data to unintended audiences (Mori et al., 2020). Furthermore, social media users are also concerned that platforms such as Tinder or WhatsApp may become targets of data breaches or share intimate files with affiliated businesses (Lutz & Ranzini, 2017).

On this basis, people report that privacy concerns are a reason to refrain from sharing personal images that disclose relationship status, sexual orientation, or specific emotions online (Miguel, 2016). A number of studies have confirmed that privacy concerns predict frequency of usage and amount of data sharing on social media (Dhir et al., 2017; Fatima et al., 2019; Jozani et al., 2020; Malik et al., 2016) to the extent of avoiding certain platforms (Choi & Sung, 2018), or social media altogether (Chen, 2018; Dhir et al., 2019; Fan et al., 2021).

Studies further revealed that young people perceive sexual online interactions with strangers as risky (Baumgartner et al., 2010a) and assessment of risk and personal vulnerability longitudinally predicts decreased engagement in sexting and other sexual online interactions with strangers (Baumgartner et al., 2010b). However, people more often sext with a romantic or sexual partner than with strangers (Klettke et al., 2014). It has not been tested whether general privacy concerns, including concerns toward the romantic partner, also motivate people to refrain from sexting. Based on the findings that privacy concerns decrease online information sharing with less sensitive content (e.g., Jozani et al., 2020), we hypothesized that individuals with high privacy concerns may be less willing to engage in sexting (*H2*)—again controlling for trait loneliness because lonely people tend to report higher privacy concerns (Lutz & Ranzini, 2017).

### Interaction of COVID-19-Related Social Isolation and Privacy Concerns

According to the privacy calculus model, privacy decisions are a cost–benefit analysis, i.e., people weigh the costs of disclosing personal information online against the benefits (Dinev & Hart, 2006). In line with that, social media users report that their privacy concerns clash with their social needs as they do care about data privacy but also want to use social media services (Hu et al., 2020; Scherini, 2020; Young & Quan-Haase, 2013). Additionally, Chen and Kim (2013) found that a high desire for gratifications on social media can outweigh privacy concerns. In line with these results, studies showed that lonely people disclose more personal data on social media (Al-Saggaf & Nielsen, 2014) although they perceive more risks (Lutz & Ranzini, 2017). For the case of sexting, young people report that fear of losing the partner leads them to engage in sexting despite concerns (Van Ouytsel et al., 2017).

Applied to sexting during the lockdown, this would mean that if people suffer from COVID-19-related social isolation, they could be more willing to sext because their desire for intimacy may override their privacy concerns. According to this theorizing, the effect of COVID-19-related social isolation on willingness to sext should be independent of privacy concerns, that is, also prevalent for those with very high privacy concerns.

Alternatively, one could argue that social isolation only drives willingness to sext when privacy concerns are low. In this case, privacy concerns would interfere with the desire for intimacy. Needless to say, the specific social isolation aimed at mitigating the spread of the COVID-19 pandemic is an exceptional situational factor and we lack research on its interaction with privacy concerns. Therefore, we explored a moderation effect of COVID-19-related social isolation and privacy concerns on willingness to engage in sexting (*RQ*).

### Gender and Age

Sexual image sharing has different implications for men and women. For women, it signifies a great risk because images of naked female bodies carry a different meaning (of being “more private” and thus more compromising) than images of naked male bodies. Due to sexual double standards, the value of an exposed female body and the reputational damage in case of a leak is disproportionally higher for women than for men (Ringrose et al., 2013, 2021). Additionally, popular media and public discourse often make women responsible for their leaked sexts and blame the victim—instead of the perpetrator—for having expected confidentiality from the interaction partner (Salter, 2016).

As a consequence, women may be more reluctant than men to share personal data (De Wolf, 2019; Dhir et al., 2017; Espinosa, 2020; Griffin et al., 2018) and have more negative expectancies of sexting (Dir et al., 2013). In line with more negative consequences for women, a recent meta-analysis concluded that women are less likely to send sexts than men (Mori et al., 2020). Therefore, we expected women to be less willing than men to engage in sexting (*H3*).

Digital communication and online sharing of personal information are relatively new phenomena and foremost adopted by younger cohorts, the so called digital natives (Anderson & Rainie, 2010). While younger people, in general, tend to react with enthusiasm to digital developments, older generations report more difficulties with and skepticism toward new technologies (Vaportzis et al., 2017; Warner-Søderholm et al., 2018). In line with that, extant research has shown that age is predictive of sexting engagement: In adults, older age has consistently been related to less engagement in sexting (De Wolf, 2019; Klettke et al., 2014; Mori et al., 2020; van Oosten & Vandenbosch, 2017; Zemmels & Khey, 2015).^2^ Therefore, we hypothesized a negative relationship between age and willingness to engage in sexting (*H4*).

## Study 1

### Method

We designed the study in accordance with the ethical guidelines of the University of Vienna and received the approval of the Institutional Review Board of the Department of Communication (ID 20200416_011). All participants gave informed consent. We collected the data for Study 1 between end of April and early May while Austria was, for the very first time, under federal lockdown due to COVID-19.

#### Participants

We recruited a young adult sample via social media because sexting is very common in young adults who are active on social media (Gesselman et al., 2020; Klettke et al., 2014; Wysocki & Childers, 2011). Young adulthood is defined as the age between 18 nd 40 years and characterized by the developmental task of balancing intimacy and isolation (Erikson, 1968). The sample consisted of 494 young adults^3^ between 18 and 40 (*M*_age_ = 24.37; *SD* = 3.88) of which 322 identified as female, 171 as male, and one person as other gender. Most (80.8%) identified as heterosexual, 13.0% as bisexual, queer, or pansexual, 4.0% as homosexual, and 2.2% preferred not to answer. About half (53.0%) were in a committed relationship and 47.0% were single. While 63.2% lived with someone else, 36.8% lived alone. Concerning adherence to measures, almost all (94.5%) agreed to currently spend more time at home due to the corona crisis, only 3.8% (19 individuals) disagreed, and 1.6% preferred not to answer.^4^ The majority of participants (56.5%) held a high school diploma, 37.7% a university degree, and 5.8% had a lower education than high school.

#### Measures

To gauge willingness to engage in sexting, we first introduced the subject with “During the corona crisis, many people are separated from their partner. Digital media provide extensive opportunities, for example using video calls, to feel close to each other.” Then we asked participants how likely it is that they engage in six online activities (from 1 = “very unlikely” to 7 = “very likely”): “send sexy text messages,” “send sexy audio messages,” “send a photo of myself in underwear or swimwear,” “send a naked photo of myself,” “send a video of myself in underwear or swimwear,” and “send a video in which I am naked” (Cronbach’s *α* = 0.86; *M* = 3.15; *SD* = 1.62). We based our items on Drouin et al. (2013) as well as on Schreurs et al. (2020).

To measure COVID-19-related social isolation, we asked participants to indicate their agreement with five statements on a scale from 1 (“fully disagree”) to 7 (“fully agree”): “I feel isolated from other people because of the coronavirus,” “It does not bother me to stay at home for a while because of the coronavirus (reverse),” “It is no problem for me to stay home until further notice because of the coronavirus” (reverse), “I feel lonely due to the coronavirus,” and “I feel like I have cabin fever.” The scale proved reliable (Cronbach’s *α* = 0.78; *M* = 2.37; *SD* = 0.91).

We measured privacy concerns using two items by Cho et al. (2010). We asked participants how likely it is on a scale from 1 (“very unlikely”) to 7 (“very likely”) that their sensitive data was abused and how likely it is that sensitive data of other people their age was abused (Cronbach’s *α* = 0.74; *M* = 4.50; *SD* = 1.50).

Moreover, participants indicated their age and self-identified gender (male/female/other). As control variables, we included trait loneliness because it is related to sexting, social isolation, and privacy concerns, respectively (Drouin & Tobin, 2014) and relationship status (Weisskirch et al., 2017). We measured trait loneliness using the revised UCLA Loneliness Scale (Russell et al., 1980) as in earlier research (e.g., Killgore et al., 2020; Tam & Chan, 2019). Out of the 20-item instrument consisting of three factors, we selected the five items with the highest factor loadings (Russell, 1996): “I feel part of a group of friends,” “There are people I feel close to,” “There are people who really understand me,” “There are people I can talk to,” and “There are people I can turn to.” Four out of these five items belonged to the subscale emotional isolation and describe the general presence of a social support circle (Döring & Bortz, 1993). The fifth emotional isolation item would have been “I can be with others if I chose to.” Due to the quarantine policy, we replaced this item with “I feel part of a group of friends” from the factor social isolation. For the purpose of this research, we deemed the presence of a social circle as more suitable than the two subscales loneliness feelings and social isolation because those would have been very similar to the construct COVID-19-related social isolation. Participants rated the five statements from 1 (“never”) to 5 (“always”). Reliability was good (Cronbach’s *α* = 0.84; *M* = 1.67; *SD* = 0.73). Participants indicated their relationship status.

#### Data Analysis

The dependent variable was negatively skewed, that is, most people reported low levels of willingness to engage in sexting. Therefore, we conducted negative binominal regression analysis using the function glm.nb from the R package MASS (Venables & Ripley, 2002). As predictors of willingness to engage in sexting, we included COVID-19-related social isolation (mean-centered), privacy concerns (mean-centered), gender, age, trait loneliness, relationship status, and the interaction between the mean-centered variables COVID-19-related social isolation and privacy concerns. The data as well as an overview of means, standard deviations, and bivariate associations are accessible under https://osf.io/zptwu/?view_only=5c3f4f0460d24141beddf1d99e7c2a8a.

### Results

All coefficients can be found in Table 1.^5^We found a significant positive effect of COVID-19-related social isolation on willingness to engage in sexting, controlling for individuals’ trait loneliness (*b* = 0.06; *p* = .019) which lends support to H1: The more participants suffered from social isolation during the lockdown, the more willing they were to engage in sexting. As expected in H2, people with higher privacy concerns reported a significantly lower willingness to engage in sexting (*b* = -0.04; *p* = .024). There was, however, no significant interaction of COVID-19-related social isolation and privacy concerns on willingness to engage in sexting (*b* = -0.02; *p* = .304; *RQ*).

**Table 1:** Negative binomial regression predicting sexting willingness in Study 1 (young adults)

Predictor	*B*	SE B	*Z*	*p*
COVID-19-related Social Isolation	0.06	0.03	2.34	.019
Privacy concerns	− 0.04	0.02	− 2.26	.024
Gender (female)	0.03	0.05	0.55	.583
Age	0.00	0.01	1.10	.271
Trait loneliness	− 0.07	0.04	− 1.93	.054
Relationship status	0.01	0.05	0.28	.779
COVID-19-related Social Isolation × Privacy Concerns	− 0.02	0.02	− 1.03	.304

Concerning demographics, we found no effect of gender (*b* = − 0.03; *p* = .583) or age (*b* = 0.00; *p* = .271) on willingness to engage in sexting, thus no support for H3 or H4.

### Discussion

People report to sext in order to satisfy the need for closeness (Albury et al., 2013); and during the COVID-19-related lockdown, sexting was a common addition to people’s sexual repertoire (Lehmiller et al., 2021). In line with those findings, we found that COVID-19-related social isolation significantly predicted people’s willingness to engage in sexting. The more people suffered from the situation around the COVID-19 pandemic, the more willing were they to engage in sexting. This is in line with terror management theory (Greenberg et al., 1986; Mikulincer & Florian, 2000).

However, people with higher privacy concerns were less willing to engage in the exchange of sexy pictures, videos, or other sensitive files. This is in line with earlier research showing that privacy concerns decrease data sharing via online messengers (e.g., Jozani et al., 2020). The interaction between COVID-19-related social isolation and privacy concerns was nonsignificant. That is, even for individuals with high privacy concerns, COVID-19-related social isolation was a predictor of willingness to sext. This suggests that the need for intimacy may override the need for privacy.

We tested if individual differences in privacy concerns moderated the effect of social isolation. Yet, future panel studies should also investigate if privacy concerns mediate the effect of social isolation on willingness to engage in sexting. Findings suggest that privacy risk assessment is not a rational process but can suffer from bias due to motivated reasoning (Cho et al., 2010; Metzger & Suh, 2017). Perhaps emotional needs alter privacy risk assessment and thereby make people more willing to sext.

Concerning demographics, individuals of all genders and ages were equally willing to engage in sexting. These results were unexpected. Yet, as a key limitation, those findings result from a convenience sample of rather highly educated young adults with a rather small age range and imbalanced distribution of genders. This limits the findings’ generalizability and therefore, a replication with a more diverse sample is warranted.

## Study 2

Most sexting research focuses on adolescents although research revealed that sexting is much more prevalent in adults (Klettke et al., 2014). Despite its prevalence in adults and despite the importance of age with regard to media use, sexting in adults is severely understudied (McDaniel & Drouin, 2015; Wiederhold, 2011). To address this lacuna, we conducted a second study in a quota-based sample of adults, testing the same hypotheses and expecting the same results as in Study 1.

### Method

The Institutional Review Board of University of Vienna’s Department of Communication approved our online survey (ID 20200321_009). All participants provided their informed consent. Data was collected during the very first lockdown in Austria in early May 2020.

#### Participants

A private polling company recruited a quota-based sample of 437 adults between 18 and 73 (*M*_age_ = 41.99; *SD* = 13.91). About half (54.5%) were female and 45.5% were male. Most (92.9%) identified as heterosexual, 1.8% as bisexual, queer, or pansexual, 1.4% as homosexual, and 3.9% preferred not to answer. Most (73.0%) were in a relationship, 24.9% were single, and 2.1% preferred not to answer. Most (79.2%) lived with someone else, while 20.8% lived alone. The majority of participants (63.6%) held a high school diploma, 33.6% a university degree, and 2.7% had a lower education than high school.

#### Measures

To assess willingness to engage in sexting, we again introduced the subject with “During the corona crisis, many people are separated from their partner. Digital media provide extensive opportunities, for example using video calls, to feel close to each other.” Then we asked participants how likely it is that they engage in the following online activities (from 1 = “very unlikely” to 7 = “very likely”). We summarized the six items of the first survey (adapted from Drouin et al., 2013; Schreurs et al., 2020) into three items: “send a sexy text or audio message,” “send a photo or video in underwear or swimwear,” and “send a naked photo or video” (Cronbach’s *α* = 0.79; *M* = 1.71; *SD* = 1.25).

All other measures were identical to Study 1: Our items for COVID-19-related social isolation proved reliable (Cronbach’s *α* = 0.78; *M* = 2.84, *SD* = 0.97). Russel’s UCLA loneliness scale (1996) demonstrated excellent reliability (Cronbach’s *α* = 0.91; *M* = 1.69; *SD* = 0.82), as well as the items by Cho et al. (2010) to measure privacy concerns (Cronbach’s *α* = 0.92; *M* = 4.65; *SD* = 1.73). We further assessed age in years, gender (male/female/other), relationship status (single/ in relationship).

#### Data Analysis

We conducted negative binominal regression analyses using the function glm.nb (Venables & Ripley, 2002) because willingness to engage in sexting was negatively skewed. We included COVID-19-related social isolation (mean-centered), privacy concerns (mean-centered), gender, age, trait loneliness, relationship status, and the interaction between the mean-centered variables COVID-19-related social isolation and privacy concerns as predictors of willingness to engage in sexting. The data as well as an overview of means, standard deviations, and bivariate associations are accessible under https://osf.io/zptwu/?view_only=5c3f4f0460d24141beddf1d99e7c2a8a.

### Results

All regression coefficients can be found in Table 2. 6 As expected in H1, we found a significant effect of COVID-19-related social isolation on willingness to engage in sexting, independent of individuals’ trait loneliness (*b* = 0.12; *p* = .005). We found, however, no support for H2: Privacy concerns were not predictive of willingness to engage in sexting (*b* = -0.03; *p* = .140). Moreover, there was no significant interaction of COVID-19-related social isolation and privacy concerns on willingness to engage in sexting (*b* = − 0.01; *p* = .834; *RQ*).

Concerning demographics, we found no effect of gender (*b* = − 0.14; *p* = .074; *H3*) but a negative effect of age (*b* = − 0.01; *p* < .001; *H4*). Women and men were equally willing to engage in sexting, but with increasing age, people were less willing to engage in sexting.

**Table 2:** Negative binomial regression predicting sexting willingness in Study 2 (Quota-based.sample).

Predictor	*B*	SE B	*Z*	*p*
COVID-19-related Social Isolation	0.12	0.04	2.78	.005
Privacy concerns	− 0.03	0.02	− 1.48	.140
Gender (female)	− 0.14	0.08	− 1.79	.074
Age	− 0.01	0.00	− 5.02	< .001
Trait Loneliness	− 0.03	0.05	− 0.52	.606
Relationship Status	− 0.02	0.09	− 0.24	.810
COVID-19-related Social Isolation × Privacy Concerns	− 0.01	0.02	− 0.21	.834

### Discussion

As in Study 2, COVID-19-related social isolation positively predicted willingness to engage in sexting. The effect was even more pronounced in the more diverse sample—even though participants in this second study felt relatively less isolated. The finding that people who suffered more from the COVID-19 situation were more willing to engage in sexting supports the tenet of terror management theory that people seek intimacy as a fear and stress relief in times of (pandemic-related) existential anxieties (Greenberg et al., 1986; Mikulincer & Florian, 2000).

However, Study 2 with, on average, older participants did not replicate the negative effect of privacy concerns found in Study 1. Participants in Study 2 were indeed more concerned about potential abuse of their sensitive data (*M* = 4.65; *SD* = 1.73 on a seven-point scale) compared to the young adult participants in Study 1 (*M* = 4.50; *SD* = 1.50). However, these higher privacy concerns did not translate into a lower willingness to engage in sexting. This is in contrast to previous research showing that the effect of privacy concerns on social media engagement is stronger in older cohorts than in young adults (Jozani et al., 2020).

One could assume that we did not find a negative effect of privacy concerns because people in Study 2 were already very unwilling to engage in sexting (*M* = 1.71; *SD* = 1.25 on a seven-point scale) out of other reasons. For participants of, on average, 42 years old, who are not digital natives, factors such as norms and lower technical affinity may have hindered them from sexting. As a consequence, privacy concerns are not a hindering factor in this age group.

Furthermore, the interaction between COVID-19-related social isolation and privacy concerns was nonsignificant. The effect of social isolation on willingness to engage in sexting was independent of people’s privacy concerns. Even in those with high concerns, their need for privacy did not interfere with the positive effect of social isolation on willingness to engage in sexting. Privacy concerns independently did not make people less willing to engage in sexting in Study 2, and they did not even moderate the positive effect of social isolation. This suggests that during the COVID-19-related lockdown, social needs were more relevant than privacy concerns.

Concerning gender, we expected that women would be less willing to engage in sexting because, due to sexual double standards, women must fear much harsher social consequences than men in case of a leaked sext (Ringrose et al., 2013; Salter, 2016). However, similar to Study 1, we found no effect of gender on willingness to engage in sexting in a sample with a balanced gender distribution. This is at odds with a recent meta-analysis concluding that women are less likely to send sexts than men (Mori et al., 2020). It may be that, during the pandemic, emotional rewards overrode the fear of negative sexting consequences that have been shown to discourage women more than men from sexting. Yet, research is not conclusive on the matter. An earlier review (Klettke et al., 2014) had found that women send more sexts than men. It remains an open question under which circumstances which women are more, less, or equally willing to engage in sexting compared to men.

Using a sample with a wide age range, we found a negative effect of age, comparable to previous research (De Wolf, 2019; Drouin & Landgraff, 2012; Klettke et al., 2014; Lehmiller et al., 2021; Mori et al., 2020; van Oosten & Vandenbosch, 2017; Zemmels & Khey, 2015). Together, our findings emphasize the importance of cross-validating models in different samples with diverse compositions.

## General Discussion

In spring 2020, the novel coronavirus (COVID-19) pandemic hit the Western world and caused an unprecedented public health and economic crisis as well as collective trauma. All of a sudden, news about infections and deaths dominated the media, obligatory face masks characterized the public sphere, and many governments closed their national borders. In Austria, the government issued a strict federal lockdown restricting all movement on March 16 (Deutsche Welle, 2021). Restaurants and hotels had to close, the police enforced adherence to the lockdown rules, and nobody could foresee how long the situation would last.

In situations of insecurity and existential anxiety, humans use intimacy as a coping strategy, according to terror management theory (Mikulincer & Florian, 2000). Yet, fear of contagion as well as limited opportunities led to a sharp decrease in physical intimacy, even within couples (Ibarra et al., 2020). During the lockdown, many people resorted to technology-based forms of intimacy such as sexting (e.g., Ballester-Arnal et al., 2020). Yet, it was hitherto unknown whether feelings of social isolation increased people’s willingness to sext.

The present research contributes to the field by investigating which factors predicted willingness to engage in sexting during the COVID-19-related lockdown. In both studies, we found a robust effect of COVID-19-related social isolation on willingness to engage in sexting, independent of individuals’ trait loneliness. This finding lends support to terror management theory (Greenberg et al., 1986), according to which people engage not only in health behaviors but also seek intimacy as a relief for (pandemic-related) existential anxieties (Mikulincer & Florian, 2000). This is the first study to show that the more individuals suffered from the measures to mitigate the spread of COVID-19, the more willing they were to engage in technology-mediated sexual exchange. The fact that the effect of COVID-19-related social isolation predicted willingness to engage in sexting over and above trait loneliness emphasizes the relevance of situational as compared to dispositional factors on sexual behavior. During this extraordinary and unprecedented time, situation was more predictive than personality.

One could theorize that the effect of COVID-19-related social isolation on willingness to engage in sexting would only be present or would be more pronounced in people who did not have a partner during the lockdown (Lehmiller et al., 2021), that is, moderated by relationship status. Yet, the main effect of COVID-19-related social isolation was significant while controlling for relationship status (and the control variable was nonsignificant). So, both singles and individuals in relationships were willing to use sexting as a coping strategy for their feelings of isolation.

Studies showed that privacy concerns can influence information exchange on social media (e.g., Jozani et al., 2020). In line with those findings, higher privacy concerns predicted a lower willingness to engage in sexting in Study 1 (young adult sample). However, this effect could not be replicated in Study 2 (quota-based sample), suggesting that privacy concerns might not be relevant for sexting in older adults because other more influential factors such as norms already prevent older cohorts from sexting. Future (qualitative) research should find out which factors, in fact, influence older adults’ willingness to engage in sexting because sexting in adults is severely understudied (McDaniel & Drouin, 2015; Wiederhold, 2011).

According to the privacy calculus model, people’s willingness to share data online is the result of a cost benefit analysis (Dinev & Hart, 2006). Drawing from this model, we tested whether the effect of social isolation on willingness to share sensitive sexual data was moderated by how much individuals valued data privacy (i.e., level of privacy concerns). We found that privacy concerns did not interfere with the effect of social isolation on willingness to engage in sexting in either study. The nonsignificant interaction underscores the importance and robustness of social isolation for digital sexual exchange: Under conditions of social isolation, even individuals with high concerns may be willing to sacrifice their privacy in favor of connection. Our finding is in line with earlier research revealing that when social needs are salient, individuals share data despite severe privacy concerns (e.g., Chen & Kim, 2013; Van Ouytsel et al., 2017). Eventually, it seems more important for humans to be connected than to set boundaries.

Concerning demographics, we found no effect of gender in either study, so during the lockdown, men and women were equally willing to engage in sexting despite potentially higher reputational damage for women (Ringrose et al., 2013, 2021). Despite sexual double standards that could discourage women from sexting, we found women were just as willing to sext during the lockdown as men. It may be that in this particular situation, women perceived rewards to exceed potential costs.

In Study 2, age was negatively associated with willingness to engage in sexting, comparable to previous sexting research (Klettke et al., 2014; Mori et al., 2020). The negative effect of age is in line with the finding that younger age predicted sexual exploration during the lockdown (Lehmiller et al., 2021) and with findings that younger populations generally endorse technology with more enthusiasm than older generations (Vaportzis, 2017; Warner-Søderholm et al., 2018). Due to the smaller age range in Study 1, it seems reasonable that the effect was not present in the younger sample.

The present research has some notable limitations. First, both studies were cross-sectional in nature which prevents inferences about timely order and causality. Prior research has associated sexting with depressed mood and anxiety (Klettke et al., 2014). So, theoretically, COVID-19-related social isolation might not have predicted sexting, but sexting made people suffer more from social isolation. As a remedial measure, we controlled for trait loneliness to factor out the influence of dispositional factors. However, it would be interesting to examine how the relationship evolves over time: Does sexting fulfill its intended goal and successfully establish an intimate connection? This is questionable because correlational research associates sexting with negative (Benotsch et al., 2013) and positive correlates (Parker et al., 2013). Much needed longitudinal research focuses exclusively on adolescents and could link sexting to bullying over time (Van Ouytsel et al., 2019) but not to well-being (Burić et al., 2021). Future research should address this gap and investigate sexting outcomes in adults. Understanding potential reciprocal (self-reinforcing) relationships would necessitate multiple waves of data collection. Although we exclusively focused on the active sending of sexts, future research could investigate how active and passive sexting relates to well-being.

Another methodological limitation is that based on prior research (Sole, 2007), we assumed an increased mortality salience as a result of the COVID-19 pandemic and therefore an increased need for (sexual) intimacy. We did, however, not measure (pandemic-based) mortality salience. Future research should measure or manipulate mortality salience to investigate if social isolation following mortality salience increases the willingness to engage in sexting. Also, for Study 2, we do not know to what extent participants adhered to the measures and how this influenced the results.

Since this study was conducted in the very first lockdown, there was no validated instrument available for the novel construct COVID-19-related social isolation. Future research should replicate our findings on COVID-19-related social isolation with a validated scale. Moreover, when measuring sexting willingness, other researchers have asked for participants’ likelihood of engaging in sexting under certain hypothetical risk-conducive situations (e.g., Schreurs et al., 2020; van Oosten & Vandenbosch, 2017, 2020) assuming that participants are not in those risk-conducive (social) situations while filling in the questionnaire. This was different in our study because we asked for likelihood of engaging in sexting during the lockdown, which was, at the time, not a hypothetical but a real situation for participants. We still refer to this as behavioral willingness because behavioral willingness is defined as “perceived likelihood of spontaneously engaging in the behavior due to social factors” (Gerrard et al., 2005, p. 306). We would see the lockdown as such conducive social factor. However, one could also argue for the terms perceived likelihood to sext (or behavioral expectation). This conceptual ambiguity is a result of the rare situation that all participants are known to be in a conducive situation during data collection.

### Conclusion

The more isolated people felt during the lockdown the higher was their willingness to engage in sexting. The fact that people react to social isolation with sexting suggests that sexting fulfills a psychological function. Exchanging intimate information may serve as an adaptive coping mechanism under difficult circumstances. Just like the couple in *Love in the time of Cholera* exchanged love letters and messages via telegraph to cope with the solitude, “love in the time of corona” may mean the exchange of erotic pictures to cope with feelings of social isolation.

Naturally, making oneself vulnerable always carries the risk of being exposed by untrustworthy interaction partners, online and offline. Yet, it is especially this vulnerability that establishes trust and connection (Amundsen, 2020). So, instead of demonizing sexting as such, research should begin to investigate its functions. Policy makers, however, should hold offenders accountable and protect vulnerable parties from exploitation.

## Data Availability

Both data sets are available on Open Science Framework.

## Footnotes

1 The response to an imminent danger would be fear. As a response to concrete fear of infection or death, we would expect directly related (“proximal”) protection behaviors, on the basis of terror management theory. The purpose of fear is to mobilize all energy toward the immediate threat, so it narrows people’s behavioral repertoire (most extremely to just fight or flight reactions; Presti et al., 2020). There are studies on the influence of COVID-19-related imminent fear and broader anxiety; these find that fear and anxiety decrease all kinds of sexual behaviors (Ibarra et al., 2020; Schiavi et al., 2020). Since sexting is a relatively new, rather non-standard (sexual) behavior, we would expect sexting to decrease as a result of COVID-19-related fear.

2 In minors, however, sexting prevalence increases with age (Madigan et al., 2018).

3 We excluded five older adults between 49 and 62 because they were not our target group, but this did not affect any results.

4 Controlling for adherence to measures did not change any results significantly.

5 In Study 1, 63.2% lived with someone else and 36.8% lived alone. Including living situation as a control variable had no effect on willingness to engage in sexting and did not change the effect of other variables. Coefficients of additional analyses can be found here https://osf.io/zptwu/?view_only=5c3f4f0460d24141beddf1d99e7c2a8a.

6 In Study 2, 79.2% reported to live with someone else and 20.8% lived alone. Including this variable did not change the results significantly although it did have a significant negative effect on the willingness to engage in sexting. Coefficients of additional analyses can be found here https://osf.io/zptwu/?view_only=5c3f4f0460d24141beddf1d99e7c2a8a.
